# New Insights Into Immunomodulatory Strategies for Drug Hypersensitivity Reactions: An EAACI Task Force Report

**DOI:** 10.1002/clt2.70195

**Published:** 2026-08-13

**Authors:** Ana Maria Copaescu, Valentina Gueli, Jitesh Chahuan, Waad Albalawi, Giovanna Sfriso, Mattia Giovannini, Leticia de las Vecillas, Aspasia Karavelia, Marina Labella, Ruben Fernandez‐Santamaria, Francesca Mori, Lene H. Garvey, Patrizia Bonadonna, Maria Jose Torres

**Affiliations:** ^1^ The Research Institute of the McGill University Health Centre McGill University McGill University Health Centre (MUHC) Montreal Quebec Canada; ^2^ Department of Clinical Immunology and Allergy McGill University Health Centre Montréal Quebec Canada; ^3^ Department of Drug and Antibiotic Allergy Services Austin Health Heidelberg Australia; ^4^ Allergy Unit University Hospital of Verona Policlinico G.B. Rossi Verona Italy; ^5^ School of Basic and Medical Biosciences & KHP Centre for Translational Medicine, King’s College London Guy’s Hospital St. John’s Institute of Dermatology London UK; ^6^ Department of Medicine University of Verona Verona Italy; ^7^ Allergy Unit Meyer Children’s Hospital IRCCS Florence Italy; ^8^ Department of Health Sciences University of Florence Florence Italy; ^9^ Department of Allergy Hospital La Paz Institute for Health Research (IdiPAZ) Madrid Spain; ^10^ ENT Department General Hospital of Kalamata Kalamata Greece; ^11^ Allergy Unit Hospital Regional Universitario de Málaga Málaga Spain; ^12^ Precision Allergy Translational Hub (PATH) IBIMA‐Plataforma Málaga Spain; ^13^ Allergy and Immunology Department IIS‐Fundacion Jimenez Diaz Hospital Universitario Fundación Jiménez Díaz Madrid Spain; ^14^ Allergy Clinic, Department of Dermatology and Allergy Herlev and Gentofte University Hospital and Department of Clinical Medicine University of Copenhagen Copenhagen Denmark; ^15^ Allergy Unit San Bortolo Hospital Ulss8 Vicenza Italy; ^16^ Departamento de Medicina, Facultad de Medicina Universidad de Málaga Málaga Spain

**Keywords:** biologics, biomarkers, desensitization, drug hypersensitivity reactions, immunomodulatory strategies

## Abstract

Drug hypersensitivity reactions (DHRs) are a global health concern and remain challenging to investigate owing to the heterogeneity of endotypes and phenotypes, as well as the lack of standardized biomarkers. Until recently, strict drug avoidance was the only management strategy for severe DHRs. However, the development of novel therapeutic options, including biologics and drug desensitization protocols, has shown promising results, particularly when no alternatives exist. In parallel, the search for reliable biomarkers for diagnosis and monitoring immunomodulatory strategies is essential to improve patient care. Advances in the algorithms and digital tools also offer opportunities for enhanced risk stratification and more efficient delabeling of patients who may be incorrectly classified as allergic. In this review, we summarize recent advances in the classification of DHRs, discuss current and emerging immunomodulatory strategies for their management, and highlight relevant biomarkers, emphasizing the need to integrate them into clinical practice.

## 1 Introduction

The current global burden of drug hypersensitivity reactions (DHRs) remains uncertain, as most data rely on self‐reported allergy labels rather than confirmed diagnoses [1, 2]. Approximately 10% of the general population interacting with healthcare systems report a drug allergy record in their medical history, with penicillin being the most frequently documented [3]. DHR accounts for an estimated 1%–2% of hospital admissions, up to 14% of emergency department visits, and 15%–20% of hospitalisations [1]. Both underdiagnosis and overdiagnosis are common in DHRs, primarily due to the lack of standardized definitions, validated biomarkers, and harmonized diagnostic strategies [4, 5, 6]. Inaccurate drug allergy labels frequently deprive patients of first‐line therapies, compromise treatment efficacy, and negatively affect quality of life and survival, particularly in populations requiring repeated or prolonged drug exposure, such as in patients with chronic inflammatory diseases, cancer, or inflammatory conditions. The impact on the healthcare system is considerable, as DHR labels are most commonly concerning antibiotics and associated with increased use of alternative, broader‐spectrum, increasingly toxic, and expensive antibiotics, contributing to antimicrobial resistance, higher rates of healthcare‐associated infections, prolonged hospital stays, and increased healthcare costs [7, 8, 9, 10, 11, 12]. These data highlight DHRs as a major clinical and public health challenge, underscoring the need for improved documentation, accurate diagnosis, and appropriate delabelling strategies to optimize patient care and healthcare resource utilization [13, 14, 15, 16, 17].

## 2 Classification of Drug Hypersensitivity Reactions

The identification and classification of distinct endotypes of DHRs are essential for a greater understanding of the specific mechanisms underlying the pathogenesis of these complex reactions. It is essential for accurate diagnosis, risk stratification, and rational intervention.

In 1963, Gell and Coombs proposed a classification of allergic reactions [18], that although somewhat simplistic, useful in clinical practice. This classification categorized allergic reactions into four types, based primarily on the pathophysiology of the reaction:

1. Type I (IgE‐mediated) with crucial involvement of mast cell/basophil activation, and is associated with clinical phenotypes, such as urticaria, angioedema, or anaphylaxis.
2. Type II (cytotoxic antibody), mediated mainly by Immunoglobulin (Ig) IgG/IgM against drug‐modified cell targets, resulting in cytopenia or organ injury.
3. Type III (immune complex), in which IgG‐antigen complexes and complement activation were the main drivers, with clinical phenotypes such as serum‐sickness‐like reactions or vasculitis.
4. Type IV (T‐cell mediated delayed reactions). This can be subclassified into IVa (T helper (Th)1/macrophage), IVb (Th2/eosinophil), IVc (CD8^+^ cytotoxic), and, IVd (neutrophil‐dominant) [19].

Nevertheless, since 1963, significant advances have been made in understanding the pathomechanisms involved in allergic reactions. These advances mean that the traditional classification of DHRs does not fit all allergic reactions, mainly non‐immunologically mediated.

Considering the foregoing, the European Academy of Allergy and Clinical Immunology (EAACI) proposed an updated classification that expands the currently known mechanisms to seven types, although it is not yet entirely suitable for classifying the DHRS: antibody‐mediated (types I–III), cell‐mediated (IVa–IVc), including pharmacological interactions (p–i concept), tissue‐driven (V–VI) direct cellular and inflammatory response to chemicals (VII) [20] (Table 1).

**TABLE 1:** Comparative table: Classical versus EAACI classification of drug hypersensitivity reactions.

Gell and Coombs classification	EAACI updated classification	Immune mechanism	Clinical phenotype	Drugs
Type I	Type I immediate	Immunoglobulins (IgE, IgG) with activation of mast cells/basophils	Immediate urticaria, angioedema, anaphylaxis	β‐lactams, platinum salts, proton pump inhibitors
Type II	Type II cytotoxic	Immunoglobulins (IgM, IgG) and macrophages activation	Drug induced cytopenia	β‐lactams, chemotherapeutics, NSAIDs, heparins
Type III	Type III immune complexes	Immunoglobulins (IgM, IgG). Antigen complexes, complement activation	Drug‐induced vasculitis, serum‐sickness	Sulphonamides, tetracyclines
Type IVa and type IVd	Type IVa T1	Th1, ILC1, Tc1, NK activation with IFN‐γ, TNF‐α, granzyme B and perforin release	SJS/TEN, MPE, delayed urticaria	β‐lactams, sulphonamides, anticonvulsants, xanthine oxidase inhibitors
Type IVb	Type IVb T2	Th2, ILC2, Tc2, eosinophils activation with IL‐4, IL‐5, IL‐9, IL‐13 production	DRESS	Sulphonamides, glycopeptide antibiotics, anticonvulsants
Type IVc	Type IVc T3	Th17, ILC3, Tc17	AGEP	β‐lactams, antimycotics, antimalarial drugs, anticonvulsants, antiepileptics, radiocontrast media
—	HLA/TCR interaction (altered peptide or p‐I concept).	Other possible types of type IV reactions (IVa–c)	Delayed exanthema, SCARs (DRESS, SJS/TEN)	Nucleoside analogue reverse‐transcriptase inhibitors, anticonvulsants, xanthine oxidase inhibitors
—	Type VII	Non‐immunological mechanism mediated by direct interaction with MRGPRX2, enzyme inhibition, or complement activation	Idiosyncratic/pseudoallergic reactions	NMBAs, fluoroquinolones, radiocontrast media, NSAIDs

*Note:* Summary of drug hypersensitivity reaction categories according to the classical Gell and Coombs classification and their alignment with the updated nomenclature proposed by the European Academy of allergy and clinical immunology (EAACI).

Abbreviations: AGEP, acute generalized exanthematous pustulosis; DRESS, Drug reaction with eosinophilia and systemic symptoms; IFN, Interferon; Ig, Immunoglobulin; ILC, Innate lymphoid cell; MPE, maculopapular exanthema; MRGPRX2, mas‐related G‐protein coupled receptor member X2; NSAID, non‐steroidal anti‐inflammatory drug; SCAR, severe cutaneous adverse reactions; SJS/TEN, Stevens–Johnson syndrome/toxic epidermal necrolysis; Tc, cytotoxic T cell; Th, T helper cell; TNF, tumor necrosis factor.

## 3 Pathomechanisms of Drug Hypersensitivity Reactions

### 3.1 Drug‐Immune System Interaction

Different mechanisms have been described in relation to the recognition of the drug by the immune system, including the hapten hypothesis, altered‐peptide repertoire, the PI‐concept, and other non‐immunological mechanisms.

#### 3.1.1 Hapten Hypothesis

This hypothesis has been described principally for β‐lactam antibiotics and other related drugs. It is based on the concept that many drugs and/or their reactive metabolites cannot interact directly with the immune system because of their low molecular weight [21, 22]. These compounds subsequently form covalent adducts with autologous proteins (haptenization) [23, 24], generating new antigens that can be recognized by antigen‐presenting cells (APCs) and presented to T cells [19, 25].

#### 3.1.2 The Altered Peptide Repertoire Model

This model is exemplified by the interaction between abacavir and a specific human leukocyte antigen (HLA) variant (HLA‐B*57:01) [26]. The interaction between the drug and the molecule alters its surface and, in a non‐covalent manner, causes the activation of T cells in the presence of the drug, with little or no interaction between the drug and the T cell receptor (TCR) [27].

#### 3.1.3 Pharmacological Interaction (p–i) Concept

Some drugs can bind non‐covalently and reversibly to HLA or TCR, thereby triggering direct activation of T cells [19, 28]. This interaction has been mainly described in severe cutaneous adverse reactions (SCARs) [29]. Different associations between drugs and specific HLA variants have been identified, such as HLA‐B*15:02 and carbamazepine‐induced Stevens–Johnson syndrome/toxic epidermal necrolysis (SJS/TEN) [30] or HLA‐B*58:01 and allopurinol‐induced SCARs [31, 32].

#### 3.1.4 Direct Interaction With Receptors on Effector Cells

Some immediate DHRs (IDHRs), can be triggered by a non‐immune mechanism through the direct activation of effector cells via the interaction of the drug with specific receptors, such as the mas‐related G‐protein coupled receptor member X2 (MRGPRX2) on mast cells, as has been observed with certain drugs, such as neuromuscular blocking agents (NMBAs), fluoroquinolones (FQs), and cationic amphiphilic drugs [33, 34, 35]. Nevertheless, most of these reactions have been studied in vitro, and their specific role in human DHRs remains uncertain. In addition, some drugs can directly activate effector cells via complement activation, leading to complement activation‐related pseudo‐allergy (CARPA) [36, 37].

#### 3.1.5 Drug Interaction With Enzymes

Some drugs, such as nonsteroidal anti‐inflammatory drugs (NSAIDs), trigger non‐allergic hypersensitivity reactions when the drug inhibits enzymes such as cyclooxygenase‐1 (COX‐1), resulting in the release of inflammatory mediators that can mimic the symptoms of an allergic response [38, 39].

### 3.2 Effector Phase of DHRs

#### 3.2.1 Effector Phase in IDHR

Basophils and mast cells are the main effector cells in these reactions. After activation, these cells degranulate and release granules containing inflammatory mediators, such as histamine and tryptase. The release of these compounds is responsible for the clinical symptoms of IDHRs, such as anaphylaxis or immediate urticaria. Immune‐mediated IDHRs are triggered by specific antibodies (IgE and/or IgG) produced by B cells and require a prior sensitization to the specific drug. As described above, non‐immune‐mediated reactions, such as those caused by direct interactions with receptors, enzymes, or complement activation [40, 41, 42], can also mimic the clinical phenotype of immune‐mediated IHDRs.

#### 3.2.2 Effector Phase in Delayed‐DHRs

These reactions are predominantly mediated by T cells, although other immune cells, including neutrophils, natural killer (NK) cells, or eosinophils may also contribute [43, 44]. As a result, these reactions exhibit greater heterogeneity in both phenotype and endotype, making their classification and study are more complex than those of IDHRs. Among their multiple mechanisms involved, these reactions may be driven by Th1, Th2, or Th17 cells. These subsets secrete cytokines such as interferon‐γ (IFN‐γ) and tumor necrosis factor‐α (TNF‐α), which activate macrophages [45]; eotaxin, interleukin (IL)‐13, IL‐5, and IL‐6, which promote the attraction of eosinophils [46, 47]; cytotoxic mediators such as perforin and granzyme B, or signals through the Fas–FasL‐mediated pathway that induce extensive keratinocyte apoptosis [48, 49]; and granulocyte‐macrophage colony‐stimulating factor (GM‐CSF), which promotes the recruitment, activation, and survival of neutrophils [50, 51].

## 4 Immunomodulatory Strategies for DHRs

Once the culprit drug has been identified, the standard approach is to avoid further exposure. However, substituting first‐line treatment with second‐ or third‐line alternatives instead of first‐line treatments may compromise therapeutic effectiveness, particularly in the context of chemotherapy [52, 53]. Furthermore, in some cases, the drug may also be indispensable when no suitable alternatives are available [54]. In the following section, we explore different immunomodulatory strategies for DHR.

### 4.1 Use of Corticosteroids, Antihistamines, and Supportive Care

Preventive strategies for DHRs focus on premedication and supportive care to minimize mast cell and basophil activation, cytokine release, and systemic inflammation. Antihistamines (H1 and H2 blockers) are the main prophylactic measure, given before exposure to high‐risk drugs (e.g., chemotherapies and monoclonal antibodies) to reduce itching, urticaria, and mild cardiovascular responses [55]. Corticosteroids are commonly used to suppress broader inflammatory pathways and are included in premedication protocols for infusion‐based therapies, especially when systemic symptoms have occurred in previous infusions. However, they may not always prevent adverse reactions, especially when they are severe, as with oxaliplatin, highlighting the need for a personalized approach to risk assessment and prophylaxis based on factors such as the patient's prior reaction history, the suspected mechanism of hypersensitivity, and the drug involved [7]. Drugs targeting specific symptoms, such as aspirin for prostaglandin‐mediated flushing and montelukast for bronchospasm, are also utilized [56]. Supportive care, including intravenous fluids, helps to reduce reaction severity by diluting mediators and supporting hemodynamic. This combined approach reduces the likelihood and severity of DHR during standard administration. The main supportive measures are summarized in Table 2.

**TABLE 2:** Preventive strategies for drug hypersensitivity reactions.

Strategy	Target symptoms
H1 antihistamines	Pruritus, urticaria, angioedema, histamine‐mediated symptoms
H2 antihistamines	Cutaneus and gastroinstestinal histamine‐mediated symptoms
Corticosteroids	Broad anti‐inflammatory effect, fever, chills, systemic inflammatory symptoms, antiemetic effect (prevention of nausea and vomiting, particularly with dexamethasone)
Leukotriene receptor antagonists	Prevention of bronchospasm
Aspirin/COX‐1 inhibitors	Flushing (prostaglandin‐mediated), systemic symptoms (rigor, fever)
Acetaminophen/antipyretics	Fever, chills
Opioids	Pain, rigors
Bronchodilators	Wheezing, bronchospasm
Other adjunctive medications (e.g. ondansetron, lorazepam)	Symptom‐specific support (nausea, anxiety, sedation…)
IV fluids (supportive care)	Dilution of circulating mediators, reduction of reaction severity
Fluid‐rate strategies for cytokine‐release risk	Reduction of cytokine‐release reaction intensity, hemodynamic support

### 4.2 Drug Desensitization

Drug desensitization (DD) is recommended for patients with hypersensitivity reactions to essential drugs for their condition, for which there are no equally effective alternatives, for example, chemotherapy drugs and biologics. Avoiding the use of first‐line agents in these cases can negatively affect prognosis, quality of life, and survival outcomes. DD allows for safe re‐exposure by inducing a temporary state of tolerance through the administration of incremental doses of the responsible drug in a controlled protocol. Large cohorts have shown that DD allows patients to continue therapy without compromising its efficacy or survival outcomes. Studies by Sloane et al. and Berges‐Gimeno et al. have demonstrated that patients with platinum‐based chemotherapy allergies who undergo RDD, such as with oxaliplatin or carboplatin, show survival outcomes, treatment efficacy, long‐term costs, and life expectancy comparable to non‐allergic patients receiving standard infusions [7, 55].

Standard DD protocols typically involve multi‐step, multi‐solution treatment regimens, with dose increases of 2–2.5 times every 15 min until the target therapeutic dose is reached. These protocols have been validated for both chemotherapeutic agents and monoclonal antibodies. Most desensitizations are performed using 3‐bag, 12‐step regimen, although 1‐, 2‐, and 4‐bag variants exist depending on drug characteristics, phenotype and risk stratification [56]. Safety data from over 2000 desensitizations show that 93% result in no, or only, mild reactions, while moderate‐to‐severe breakthrough symptoms occur in 7%, none of which precludes completion of treatment, and there have been no reported fatalities associated with DD protocol use [55].

In vitro IgE desensitization models have been developed to reproduce and thoroughly study the cellular and molecular mechanisms underlying the protocol. The DD of mast cells under physiological conditions results in marked hypo‐responsiveness to the desensitizing drug, with consequent inhibition of mast cell degranulation, calcium flux, and cytokine secretion, as well as suppression of both the early and late phase responses [57]. The achieved desensitization is antigen‐specific; where immune cells can still respond to an unrelated antigen, which indicates that the process does not compromise FcεRI signaling. Further studies have shown that FcεRI, IgE, and antigens are not internalized during desensitization. Desensitized mast cells retain surface IgE capable of binding the antigen but are unable to mobilize intracellular calcium and undergo exocytosis FcεRI signaling is unaffected by changes in the phosphorylation of kinases, such as Lyn, LAT, and ERK. However, calcium‐dependent events are affected, confirming the role of cytoskeletal regulation in desensitization [58]. A recent multi‐step desensitization model has confirmed that the inhibitory process is both dose‐ and time‐dependent and is partially regulated by SH2‐containing inositol phosphatase 1 (SHIP‐1). In summary, these findings demonstrate that the desensitization process is accompanied by a modulation of the signaling process, the activation of which is limited by reduced receptor‐ligand binding times and cytoskeletal rearrangements. In vitro IgE desensitization model has confirmed that the inhibitory process is both dose‐ and time‐dependent [57, 58, 59].

DD not only prevents immediate clinical reactivity but also induces measurable immunomodulatory changes affecting mast cells, basophils, and the adaptive immune response. Clinically, repeated desensitization cycles may lead to a progressive reduction in the frequency and intensity of breakthrough reactions, suggesting the induction of a more sustained hyporesponsive state beyond simple pharmacologic blockade [60]. At the effector‐cell level, DD suppresses mast cell activation by inhibiting calcium mobilization and downstream degranulation pathways, as demonstrated in vivo by the loss of skin‐test reactivity to the culprit drug after each desensitization protocol [61].

Biomarker studies further confirm the reduced activation of effector cells. In IgE‐sensitized cancer patients undergoing rapid desensitization to platinum compounds, modulation of soluble FcεRI (sFcεRI), IgE, and tryptase indicates controlled mast cell activation. Patients with higher baseline levels of sFcεRI showed lower tryptase release and fewer breakthrough reactions, suggesting a protective role for sFcεRI in blocking free IgE and preventing FcεRI cross‐linking [62]. The basophil activation test (BAT) has emerged to be a promising tool for delabelling and endotyping IDHRs to platinum salts and taxane [63]. It may also be useful for monitoring DD, as reduced basophil reactivity to the drug antigen correlates with the development of clinical tolerance, as observed in patients desensitized to brentuximab vedotin [64].

In addition to immediate effector pathways, DD modulates adaptive immunity. A longitudinal analysis of desensitization to infliximab showed reductions in both anti‐drug IgE and IgG antibodies, as well as repression of drug‐specific T cell proliferation across successive DD cycles. This was associated with the onset of IL‐35‐producing regulatory T cells, indicating drug‐specific induction of immune regulation [61]. Further evidence shows that successful desensitization is associated with a significant increase in circulating levels of IL‐10, regardless of the responsible drug or reaction phenotype, further emphasizing the role of regulatory cytokines in establishing transient tolerance [65].

Overall, these findings demonstrate that DD involves a network of immunomodulatory mechanisms, including effector cell hypo‐responsiveness, alterations in soluble and cellular biomarkers, and induction of regulatory cytokines and T cells, which together attenuate reactivity and promote safe re‐exposure to culprit drugs.

### 4.3 Targeted Biological Therapies

Targeting alarmins, type 2 cytokines, and IgE has yielded variable results across several allergic diseases [66]. Therapies targeting these molecules are designed to modulate type 2 immune responses, with the potential to reduce classical allergic reactions and drug‐induced hypersensitivities [67]. The different strategies with biological therapies in DHRs are summarized in Table 3. It should be noted, however, that biologics are themselves capable of inducing hypersensitivity reactions, and this dual role, as both a therapeutic strategy and a potential trigger, warrants consideration when incorporating these agents into DHR management. As large‐molecular‐weight agents, biologics do not require haptenation to elicit an immune‐mediated reaction [91]. Reported mechanisms include IgE‐mediated immediate reactions [92], as well as IgG‐mediated reactions via low‐affinity FcγRIII receptors on basophils, macrophages, and neutrophils [93]. Non‐antibody‐dependent immediate reactions have additionally been described, including cytokine release reactions (CRR) arising from direct target‐cell activation, and CARPA, increasingly implicated in patients with rapid‐onset symptoms despite negative skin testing and undetectable drug‐specific IgE [92].

**TABLE 3:** Biological therapies for drug hypersensitivity reactions.

Monoclonal antibody	Mechanism of action	Drug hypersensitivity reactions	References
Anti‐IgE (omalizumab)	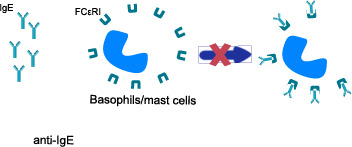 Binds free IgE → prevents IgE from binding FcεRI → downregulates FcεRI → reduces mast cell/basophil activation.	Chemotherapy‐induced hypersensitivity NSAID hypersensitivity (including N‐ERD/AERD) Insulin hypersensitivity	[68, 69, 70, 71, 72, 73]
Anti IL‐5 (mepolizumab, reslizumab)/anti IL‐5R (benralizumab)	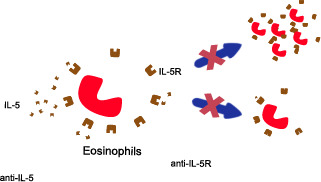 Blocks IL‐5 or IL‐5R → reduces eosinophil maturation/survival → JAK/STAT signaling inhibited.	DRESS	[74, 75, 76, 77, 78, 79, 80, 81]
Anti–IL‐4Rα (dupilumab)	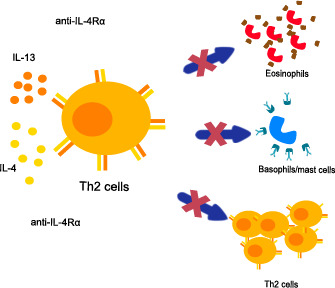 Blocks IL‐4 and IL‐13 signaling via IL‐4Rα → reduces Th2‐mediated inflammation, including eosinophils and IgE pathways.	DRESS	[47, 82, 83, 84]
Anti–TNF‐α (etanercept, infliximab)	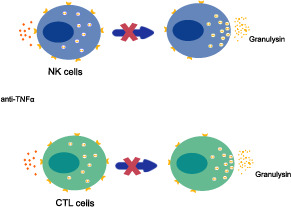 Reduces TNF‐α induced cytotoxicity → decreases granulysin release.	SJS/TEN	[85, 86, 87, 88, 89, 90]
Anti‐TSLP (tezepelumab)	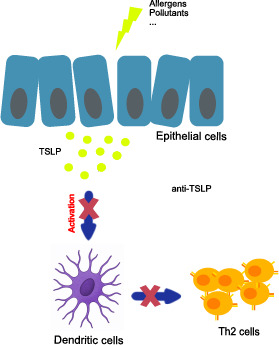 Prevents activation of dendritic cells → inhibits initiation of Th2 pathway	No clinical cases reported in DHRs yet	[66, 67]

Clinicians should therefore apply the same biomarker‐based phenotyping approaches discussed in Section 5 to distinguish a genuine hypersensitivity reaction to the biological agent itself from an expected pharmacologic or infusion‐related effect.

#### 4.3.1 Anti‐IgE

Omalizumab is a humanized IgG1 monoclonal antibody that binds to free IgE. This prevents interaction with the FcεRI receptor on mast cells and basophils, reducing IgE‐mediated activation and inducing downregulation of FcεRI.

Several case reports and case series have described the use of omalizumab in the management of DHRs, either as an adjunct to desensitization protocols or as an alternative therapeutic approach. In one case series, seven of eight patients with hypersensitivity to chemotherapy agents were able to complete their therapies using omalizumab without desensitization [68]. In another series of 8 cases, omalizumab enabled successful desensitization after failure of a 16‐step protocol, demonstrating efficacy in IgE‐mediated and mixed‐phenotype reactions [69].

Further reports have described the successful use of omalizumab in desensitization to other drugs, including NSAIDs and aspirin (particularly in NSAID‐induced respiratory disease [N‐ERD]) and insulin [70, 71, 72, 73].

#### 4.3.2 Anti‐IL‐5/IL‐5R

IL‐5 is a key cytokine in eosinophil maturation, while IL‐5R activation triggers the JAK‐STAT pathway. Monoclonal antibodies targeting IL‐5 or IL‐5R can reduce circulating eosinophils, providing a rationale for their use in eosinophil‐mediated disorders, including DRESS [74]. Currently, there is no standard treatment for DRESS, given its low incidence and variable clinical presentation. Biologics targeting IL‐5 or IL‐5R have emerged as steroid‐sparing therapies in selected DRESS patients [75, 76, 77, 78]. Gschwend et al. treated 14 patients with DRESS using anti‐IL‐5 agents. The main indications for IL‐5 blockade were therapy‐refractory disease, recurrent relapses, or severe organ dysfunction [79]. A subsequent analysis confirmed clinical improvement with biologics targeting the IL‐5 axis [80].

These findings show that anti‐IL‐5 therapies could be effective in cases of corticosteroid‐resistant DRESS and could be used as an alternative for patients at a high risk of corticosteroid‐related toxicity. The results are encouraging, but the evidence is currently limited. The optimal dosing, timing, and treatment duration of anti‐IL‐5/IL‐5R monoclonal antibodies in DRESS have yet to be defined, and large multicenter trials remain necessary.

#### 4.3.3 Anti‐IL‐4Rα

Recent studies on DRESS immune endotypes have shown significantly elevated levels of IL‐4 and IL‐13. This supports the idea that targeting the IL‐4/IL‐13 pathway could be therapeutic [47, 82].

In a small case series of two patients with DRESS, dupilumab rapidly improved symptoms and reduced steroid use. After discontinuation, a mild rebound was observed, which resolved upon restart [83]. The authors suggested a prolonged course (≥ 6 months) to prevent relapse.

Another case report described a patient with steroid‐resistant DRESS who responded quickly to dupilumab and mycophenolate mofetil following a flare‐up of cutaneous symptoms during tapering [84]. The selection of dupilumab was guided by RNA in situ hybridization, which revealed high levels of IL‐4, IL‐13, and IFN‐γ in the involved skin, thereby enabling a steroid‐sparing approach.

Anti‐IL‐4/IL‐13 biologics have shown promising potential in treating DRESS. However, current evidence is limited to case reports. There is a strong rationale to further investigate these biologics as therapeutic options for Th2‐driven cases of DRESS.

#### 4.3.4 Anti‐TNF‐α

In Stevens‐Johnsons syndrome/toxic epidermal necrolysis (SJS/TEN), TNF‐α is strongly expressed in plasma and vesicle fluid and is implicated in cytotoxic T cell‐mediated pathogenesis, probably acting as an upstream regulator of granulysin [85], one of the main effectors of keratinocyte detachment. In a randomized controlled trial of 96 patients with SJS/TEN, both TNF‐α and granulysin decreased after treatment with etanercept, suggesting that this biologic reduces granulysin by blocking the TNF‐α pathway [86]. A recent study examining the role of TNF‐α in dapsone hypersensitivity showed that, during the early phase of SCAR, TNF‐α promotes a positive feedback loop that amplifies effector CD8^+^T cell activation in SCAR. In the effector phase, TNF‐α instead modulates Treg activation [87]. These findings are important for the timely use of TNF‐α antagonists in the treatment of SCARs. The use of anti‐TNF‐α agents in SJS/TEN has also been reported in several case reports and case series in the literature [88, 89, 90].

#### 4.3.5 Anti‐TSLP

Anti‐thymic stromal lymphopoietin (TSLP) agents block this alarmin in epithelial tissues, preventing the activation of dendritic cells and the initiation of the Th2 inflammatory cascade [66, 67]. TSLP is a key driver of type‐2 immunity, however, its relevance in the immunopathogenesis of severe drug reactions remains uncertain. Nonetheless, the biological rationale is compelling, and anti‐TSLP steroid‐sparing therapy may represent a promising strategy, particularly in patients with prominent Th2‐skewed inflammation. Nevertheless, no clinical cases or studies have reported the use of anti‐TSLP biologics as targeted therapy for DHRs.

### 4.4 Regulatory Checkpoints in DHRs

The roles of regulatory T cells (Tregs) and B cells (Bregs) have been extensively investigated in the context of allergic reaction [94, 95]. Low frequency or dysfunction has been linked to allergic reactions, and its restoration during immunomodulatory strategies, such as allergen immunotherapy (AIT) significantly reduces symptoms in allergic patients [96, 97]. Nevertheless, their specific role in DHR remains unknown [98]. These cells exert their function by producing cytokines such as IL‐10, IL‐25, transforming growth factor beta (TGF‐β), or galectin‐9 (GAL‐9) [99]. In a recent study, desensitization with biologics such as rituximab and tocilizumab, increased the frequency of Treg cells, and IL‐35 production [99]. Due to the modulatory activity of these cells, it is plausible that targeting them and the molecules they produce could have a significant impact on DHR modulation, however, further research is needed to better understand their potential role in these reactions.

## 5 Biomarkers for Monitoring Immunomodulatory Efficacy in DHRs

### 5.1 Immediate DHRs

**FIGURE 1:**
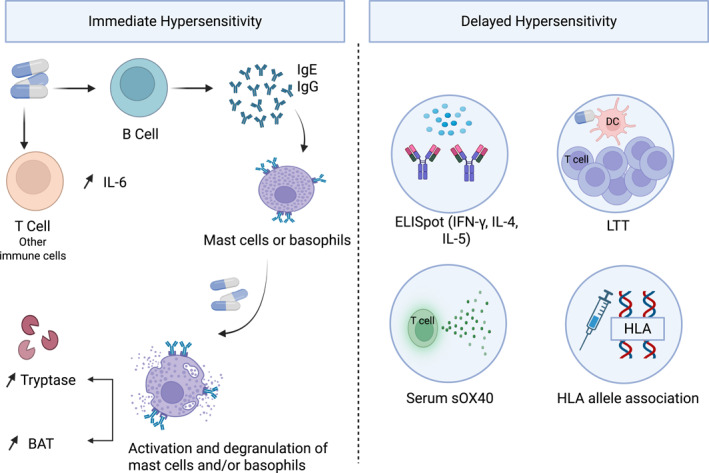
Mechanisms and biomarkers of immediate and delayed drug hypersensitivity. Immediate hypersensitivity (left panel): Following exposure to a culprit drug, B cells produce drug‐specific antibodies (IgE and/or IgG) that bind to mast cells and basophils via their respective Fc receptors (FcεRI for IgE; FcγRIII for IgG), leading to cellular activation and degranulation. This classical pathway releases preformed and newly synthesized mediators, detectable using tryptase and the basophil activation test (BAT). A distinct, increasingly recognized phenotype of immediate hypersensitivity is cytokine release syndrome (CRS), arising from direct activation of drug target cells rather than antibody‐dependent mast cell/basophil degranulation. Interleukin‐6 (IL‐6) has been established as a possible biomarker for this phenotype and is shown here as a separate pathway, distinct from the tryptase/BAT axis. Delayed hypersensitivity (right panel): T cell‐mediated mechanisms can be assessed using ELISpot (IFN‐γ, IL‐4, IL‐5), the lymphocyte transformation test (LTT), serum soluble OX40 (sOX40), and HLA allele association testing. BAT, basophil activation test; ELISpot, enzyme‐linked immunospot; HLA, human leukocyte antigen; LTT, lymphocyte transformation test. *Source:* Created with BioRender.

Drug anaphylaxis is a severe, potentially life‐threatening hypersensitivity reaction with a high economic burden that requires prompt recognition and management [100]. Laboratory confirmation of mast cell degranulation provides an adjunctive diagnostic criterion that complements detailed clinical assessment [101]. In recent years, clinical guidelines have increasingly emphasized the importance of incorporating objective biomarkers, mostly mast cell‐derived mediators, to support clinical diagnosis, particularly in cases where symptom presentation may be atypical or ambiguous [100]. These biomarkers enhance diagnostic accuracy and contribute to a better understanding of the underlying pathophysiology of drug anaphylaxis, thereby informing acute management (Figure 1) [100].

Tryptase is one such marker is. First introduced by Schwartz et al. in 1987 [102], tryptase remains the principal biomarker for detecting human mast cell activation. It is currently the most widely used laboratory marker for diagnosing immediate hypersensitivity reactions, including anaphylaxis [101]. However, the role of this marker in drug reintroduction procedures, such as DD remains unknown. As discussed in the previous section, DD is commonly used to manage immediate drug reactions to chemotherapeutic agents. In this context, some authors have explored using tryptase levels as a marker of breakthrough reactions in patients with platinum hypersensitivity reactions [103, 104]. Similarly, in another report, elevated serum tryptase levels were observed twice during rituximab desensitization in the same patient, coinciding with clinical signs of IDHR [56].

Another marker for IDHRs is drug‐specific (s)IgE. In a recent large cohort study that analyzed 9100 patients with 15,000 sIgE analyses for penicillin allergy, the authors concluded that sIgE for one or more penicillins is likely to be positive in patients with severe immediate reactions characterized by cardiovascular symptoms, angioedema, and treatment with adrenaline. In the context of platinum desensitization, total IgE was an independent predictor of breakthrough reactions [105]. In addition, as discussed previously, the use of IgE‐targeting biologics, such as omalizumab, as an adjunctive therapy during DD for IgE‐mediated hypersensitivity reactions has shown promise in enhancing patient tolerance and reducing the severity of breakthrough reactions [106, 107]. By targeting circulating IgE and downregulating FcεRI expression on effector cells such as mast cells and basophils, omalizumab may modulate the immune response and create a more favorable environment for successful desensitization [108, 109]. Preliminary studies and case reports have demonstrated encouraging outcomes; however, robust evidence from large‐scale randomized controlled trials remains insufficiently investigated [110]. Further research is essential to validate its efficacy, determine optimal dosing strategies, and assess long‐term safety and outcomes in diverse patient populations undergoing desensitization [110].

Emerging evidence suggests that cytokines, such as IL‐6 may serve as markers of immune activation and modulation, yet their utility remains underexplored [111]. For example, in patients undergoing DD, high IL‐6 levels have been associated with an increased risk of breakthrough reactions, whereas IL‐10 levels have been associated with successful tolerance induction DD [112]. A recent study demonstrated that IL‐6 signaling suppresses the differentiation of naïve CD4^+^T cells into Th2 cells, which are central to IgE‐mediated allergic responses [113]. Elevated levels of IL‐6 and CCL2 have also been associated with perioperative anaphylaxis, indicating their potential as a biomarker [114].

Among the ex vivo/in vitro tools currently available in the DHR research setting, BAT has shown interesting results. BAT is a flow cytometry‐based assay that measures basophil activation by assessing surface protein expression, most commonly CD63 and CD203c, before and after antigen stimulation. BAT detects basophil degranulation triggered by both IgE‐dependent and non‐IgE‐dependent mechanisms, and a positive test cannot distinguish between these mechanisms [115, 116]. BAT has been used for multiple agents, including β‐lactam antibiotics, NMBAs, chemotherapeutics, opiates, and biologics with variable diagnostic utility [116]. Current evidence suggests that BAT has limited diagnostic value in β‐lactam hypersensitivity [115, 117, 118]. For chemotherapeutic agents, BAT was positive in most patients with platinum allergy, and negative in all control groups, indicating high sensitivity and specificity in this population [103]. It was also positive in patients with elevated serum tryptone levels, suggesting simultaneous activation of basophils and mast cells [103]. BAT was also positive in 92% of RDD cases with breakthrough reactions, compared to 50% of RDD cases without breakthrough reactions, indicating a promising biomarker for predicting breakthrough reactions during RDD [103]. In a case report of oxaliplatin‐induced recurrent fever approximately 30 min after drug administration, with negative skin and IgE tests, the BAT was positive, and the patient tolerated desensitization, supporting a non‐specific mast/basophil‐mediated reaction [119]. The reliability of BAT is highly influenced by multiple factors, including the culprit drug class, clinical presentation, protocols, the time between the reaction and testing, and the use of immunosuppressant [116]. These findings highlight the potential role of BAT as a diagnostic and prognostic tool, while also acknowledging its limitations, such as the rate of non‐responders or the need for fresh blood, for adaptation in clinical practice.

Another diagnostic tool used for both non‐IgE‐ and IgE‐mediated reactions is the mast cell activation test (MAT), which relies on flow cytometry to quantify activation and degranulation markers of mast cells. Understanding that certain drugs can directly activate the MRGPRX2 receptor on the surface of mast cells, and that this receptor can induce direct degranulation without prior sensitization, the MAT can be an additional tool to overcome the limitations of BAT and to better understand these mechanistic pathways [115, 120]. Nevertheless, the lack of standardization, variability in protocols and markers, use of cell lines, potential for false positives/negatives, restricted availability in routine clinical practice, and the complexity of in vitro culture techniques limit its inclusion as a routine technique. Serum MRGPRX2 levels have also been measured in the context of iodinated contrast media‐induced reactions, with authors finding that it can be a potential biomarker for predicting anaphylaxis when measured 2–4 months after the reaction [121].

Despite the widespread implementation of immunomodulatory strategies, such as drug desensitization, there remains a significant gap in our understanding of the biomarkers that could guide and monitor these interventions.

### 5.2 Delayed DHRs

Delayed DHRs range in severity from mild, self‐resolving skin eruptions to SCAR [122, 123]. SCARs (e.g., SJS/TEN, DRESS, AGEP) represent some of the most serious and life‐threatening manifestations of DHRs [122, 123, 124], with possible long‐lasting T‐cell immune response [125]. Traditional in vivo diagnostic approaches pose significant risks for these patients, necessitating the development of safer, more precise alternatives. In this context, ex vivo/in vitro assays are emerging as valuable tools for evaluating immune responses in SCARs (Figure 1) [126].

The lymphocyte transformation test (LTT) assesses delayed DHRs by detecting drug‐specific T‐cell proliferation [127, 128]. LTT limitations include unavailability and variable sensitivity and specificity [43, 44, 122, 126, 128]. LTT diagnostic accuracy has been enhanced by specific phenotypes, such as DRESS [112, 122, 129, 130]. Although it has been shown that LTT can be relevant for diagnosing delayed DHRs, especially in severe SCAR, and provides relevant information on the immune mechanism involved, no evaluation has been conducted to determine its role following immunomodulation strategies.

Another research‐based tool is the enzyme‐linked immunospot (ELISpot) assay [131, 132]. The ELISpot assay is an emerging ex vivo diagnostic tool used to detect cytokine release from drug‐specific cells, most commonly IFN‐γ [132, 133, 134]. The use of other cytokines, such as IL‐4 and IL‐5, may enhance the sensitivity of ex vivo testing in DRESS patients [135, 136, 137]. Furthermore, the type of patient sample appears to play an important role, as blister fluid cells demonstrated greater sensitivity for detecting drug‐specific immune responses than peripheral blood mononuclear cells (PBMCs) in SCARs. Overall, while ELISpot offers high specificity, its performance varies with the drug, timing, and cytokine target [138]. Further validation and standardization are needed before routine clinical implementation.

In cases of DRESS, certain biomarkers, such as elevated eosinophil counts [139], atypical lymphocytosis, and human herpesvirus (HHV) reactivation [140], particularly HHV‐6 and HHV‐7, have been associated with disease activity and severity [141]. Cytokines and chemokines such as IL‐4, IL‐5, IL‐6, JAK3, STAT1, TARC, TNF‐α, and CXCL10/IP‐10 (IFN‐γ‐induced protein 10) [142] have been implicated in the pathogenesis of DRESS and may serve as biomarkers for immune activation [143]. In this context, biologics such as dupilumab [84] and omalizumab [144] have shown potential as therapeutic options for DRESS syndrome. Furthermore, serum soluble OX40 (sOX40) is a promising biomarker for the early diagnosis and severity assessment of DRESS, as its levels correlate with CD4^+^ T cell activation, cytokine profiles, and HHV‐6 reactivation [145]. Finally, a transcriptomic pilot study of 5 cases identified a six‐gene biomarker panel (STAC, GPR183, CD40, CISH, CD4, and CCL8) with high sensitivity and specificity (100% and 85.7%–100%, respectively) for diagnosing antibiotic‐associated DRESS, distinguishing it from other inflammatory conditions and supporting its potential for personalized drug hypersensitivity diagnostics [146].

For SJS/TEN, the immune mechanisms leading to widespread epidermal detachment and keratinocyte apoptosis are poorly understood [48, 147]. Recent studies focus on various signaling pathways that can provide therapeutic approaches such as the galectin‐7/TRPM2/Zn(2+)/DRP‐1 signaling pathway [148], ferritinophagy‐mediated ferroptosis, lipid metabolism, and the FSP1‐CoQ‐dependent pathway [149], NLRP3 inflammasome formation [150], Janus Kinases‐ Signal Transducers and Activators of Transcription (JAK‐STAT), Transforming Growth Factor Beta (TGF‐β), and Macrophage Migration Inhibitory Factor (MIF) pathways [123, 151]. Building on these findings, JAK inhibitors (tofacitinib, baricitinib, abrocitinib, upadacitinib) significantly reduced keratinocyte‐directed cytotoxicity in vitro and improved outcomes in mouse models [152]. Further, seven patients with TEN treated with JAK inhibitors showed rapid re‐epithelialization and full recovery, marking a breakthrough in targeted therapy for this previously untreatable condition [152].

Additionally, genetic markers, especially HLA alleles, are increasingly recognized for their utility in pre‐prescription screening and diagnostic strategies [123]. Several studies suggest an association between specific HLA‐A/B alleles and an increased risk of drug‐induced adverse reactions [123]. However, HLA‐B screening to prevent SCARs is limited by the diversity of phenotypes and the wide range of culprit drugs [123, 153].

The limited characterization of these biomarkers underscores the urgent need for systematic research to elucidate their relevance, reliability, and clinical applicability in DHRs. To address this, future research priorities should include validating tryptase and other cytokines as monitoring tools, integrating multi‐omics approaches, and developing standardized biomarker panels tailored to specific drug classes and patient populations. Techniques such as BAT, MAT, LTT, ELISpot, and T‐cell activation markers offer mechanistic insights and potential diagnostic utility [126]. However, their clinical integration remains limited due to challenges in standardization and validation.

## 6 Future Directions

**FIGURE 2:**
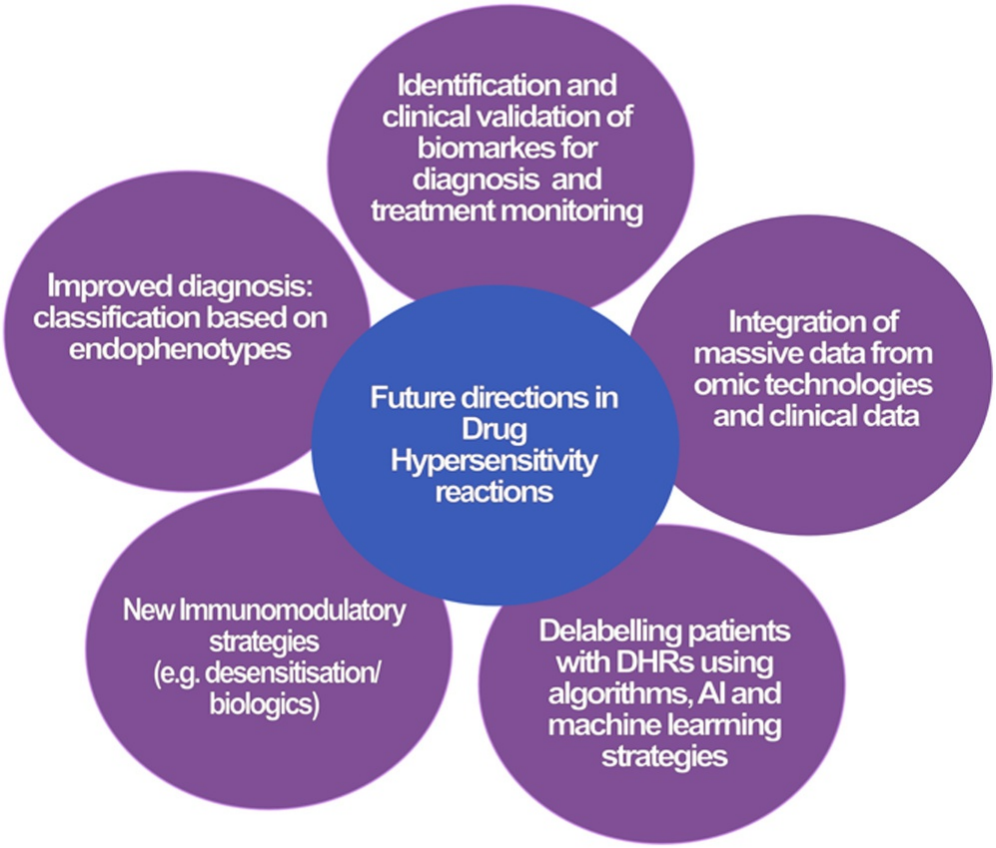
Future direction in drug hypersensitivity reactions.

The diagnosis and investigation of DHRs remain challenging because of the complexity of reactions, the heterogeneity of endotypes, which can lead to indistinguishable phenotypes, and the lack of validated, sensitive, and specific biomarkers. Therefore, personalized medicine is essential to achieve an accurate diagnosis, move toward classification of endo‐phenotype and enable identification of the most appropriate immunomodulatory strategy. To achieve this, it is essential to improve, standardize, and validate in vivo and in vitro/ex vivo biomarkers for both immediate [93, 154, 155, 156] and delayed DHR [14, 155, 157] (Figure 2).

In recent decades, advances in artificial intelligence (AI) and machine learning algorithms have been explored to reduce the number of individuals mistakenly labeled as allergic in an effective, economical, and safe manner [158, 159]. These approaches help to define patient risk‐stratification [158, 160, 161]. Algorithms can be used by non‐specialized allergists (e.g., in primary care settings [162] or by pharmacists [163]). These stratification tools can help identify low‐risk patients, which can facilitate the decision‐making process to directly remove the label or safely refer to drug provocation test, thereby reducing cost and time in both the adult and pediatric populations [13, 14, 164]. However, these algorithms and digital tools must be validated in different countries, drugs, and age groups.

The emergence of omics technologies has proven relevant to the study of allergic diseases, as they provide valuable information on the identification and/or validation of biomarkers, as well as the description of new potential therapeutic targets, improving patient stratification, accurate prognosis, and monitoring of specific treatment in the context of precision medicine [165, 166, 167]. In DHRs, metabolomics is of particular interest, as some drugs are rapidly metabolized and degraded, making the identification of reactive metabolites essential for identifying the specific trigger and mechanism of DHRs [21, 23, 25, 168, 169, 170, 171, 172]. Furthermore, new insights in proteomics have enabled a better understanding of the complex interactions between drugs and protein [22, 23, 167, 173, 174, 175, 176, 177, 178]. In addition, the development of genomics has made it possible to associate different variants, mainly HLA, with a higher risk of developing DHRs [179], and specifically to β‐lactams [180, 181].

In recent decades, advances in biologics have opened the door to new therapeutic options for treating various allergic diseases, especially difficult‐to‐treat hypersensitivities. In the context of DHRs, although there are currently no biologics approved for clinical use, research has been conducted to evaluate their potential role in desensitization to improve outcomes, clinical efficacy, and reduce side effects [182]. In addition, omalizumab has been investigated to manage hypersensitivity reactions to NSAIDs [108] and aspirin [182, 183], although further research is warranted.

Currently, desensitization protocols are now an increasingly used and cost‐effective strategy when the culprit drug is essential, and no suitable alternatives are available, particularly in the context of chemotherapeutic agents. Several biomarkers are currently under investigation and have shown promising results [63, 184, 185, 186], however, further refinement and validation are required to reliably predict DHRs and treatment outcomes.

In summary, although significant progress has been made in understanding and managing DHRs, substantial challenges remain in achieving accurate diagnoses and effective treatments. Continued efforts to validate biomarkers, integrate omics technologies, and apply AI‐driven algorithms and digital risk‐stratification tools will be essential to advance precision medicine and treatment monitoring. Ultimately, improving diagnostic accuracy and personalizing therapeutic strategies will reduce mislabeling, optimize patient outcomes, and lessen the healthcare burden associated with DHRs.

## Author Contributions

**Ana Maria Copaescu:** writing – original draft, writing – review and editing. **Valentina Gueli:** writing – original draft, writing – review and editing. **Jitesh Chahuan:** writing – original draft, writing – review and editing. **Waad Albalawi:** writing – original draft, writing – review and editing. **Giovanna Sfriso:** writing – original draft, writing – review and editing. **Mattia Giovannini:** conceptualization, writing – original draft, writing – review and editing. **Leticia de las Vecillas:** conceptualization, writing – review and editing, writing – original draft. **Aspasia Karavelia:** conceptualization, writing – original draft, writing – review and editing. **Marina Labella:** writing – original draft, writing – review and editing. **Ruben Fernandez‐Santamaria:** conceptualization, writing – original draft, writing – review and editing. **Francesca Mori:** writing – review and editing, supervision. **Lene H. Garvey:** supervision, writing – review and editing. **Patrizia Bonadonna:** writing – review and editing, supervision. **Maria Jose Torres:** writing – review and editing, supervision.

## Ethics Statement

The authors have nothing to report.

## Conflicts of Interest

J.C. reports previous employment on a grant provided by Epsilogen Ltd. M.G. reports personal fees from Sanofi and Thermo Fisher Scientific. The remaining authors declare no conflicts of interest related to this publication. All authors have reviewed and approved this manuscript.

## Data Availability

Data sharing not applicable to this article as no datasets were generated or analysed during the current study.
