# Correction to “New Insights Into Immunomodulatory Strategies for Drug Hypersensitivity Reactions: An EAACI Task Force Report”

**DOI:** 10.1002/clt2.70199

**Published:** 2026-09-07

A. M. Copaescu, V. Gueli, J. Chahuan, et al., “New Insights Into Immunomodulatory Strategies for Drug Hypersensitivity Reactions: An EAACI Task Force Report,” *Clinical and Translational Allergy* (2026): e70195, https://doi.org/10.1002/clt2.70195.

In the author list, the name of one of the co‐authors was published incorrectly as “Jitesh Chahuan”. The correct author name is “Jitesh Chauhan”. This error has been corrected.

We apologize for this error.

